# Computational discovery of fenugreek–paitan–turmeric (FPT) bioactive compounds targeting SGLT2 and DPP-4 for glucose homeostasis regulation

**DOI:** 10.1016/j.jtumed.2026.05.008

**Published:** 2026-06-09

**Authors:** Gita S. Prihanti, Anung P. Illahika, Noviana D. Lestari

**Affiliations:** a Department of Medical Education, Faculty of Medicine, University of Muhammadiyah Malang, Malang, Indonesia; b Department of Anatomy, Faculty of Medicine, University of Muhammadiyah Malang, Malang, Indonesia; c Faculty of Medicine, University of Muhammadiyah Malang, Malang, Indonesia

**Keywords:** Computational analyses, Diabetes mellitus, DPP-4, Glucose homeostasis, SGLT2

## Abstract

**Objectives:**

This study aimed to evaluate the bioactive compounds identified in fenugreek–paitan–turmeric (FPT) by liquid chromatography-high resolution mass spectrometry analysis and their interactions with dipeptidyl peptidase-4 (DPP-4) and sodium–glucose cotransporter-2 (SGLT2), which are associated with glucose regulation.

**Methods:**

Computational analyses were conducted, including molecular docking to screen potential compounds and molecular dynamics simulations, where 250 ns trajectories were evaluated to assess complex stability, and further validated using molecular mechanics Poisson–Boltzmann surface area (MM/PBSA), dynamic cross-correlation matrix (DCCM), and principal component analysis (PCA).

**Results:**

Molecular docking identified (+)-ar-turmerone and β-carboline-3-carboxylic acid as compounds with moderate binding affinities toward DPP-4 (−6.9 kcal/mol) and SGLT2 (−8.6 kcal/mol), respectively. Molecular dynamics simulations indicated a more favorable interaction with SGLT2 than DPP-4, although structural fluctuations were observed, particularly after 100 ns. MM/PBSA analysis identified a favorable binding free energy for SGLT2 of −42.135 kJ/mol, whereas DPP-4 had a positive value of +53.907 kJ/mol, suggesting unfavorable binding. These findings were supported by DCCM analyses and PCA, which indicated more constrained motions in the SGLT2 complex relative to DPP-4.

**Conclusions:**

In this study, preliminary computational insights were obtained into the interactions of FPT-derived compounds with SGLT2 and DPP-4. The results suggested a more favorable interaction with SGLT2, whereas the binding to DPP-4 appeared less stable. Further experimental validations, including in vitro and in vivo studies, are needed to validate their efficacy in the management and pathogenesis of diabetes.

## Introduction

Diabetes is a serious global metabolic disease and its prevalence is increasing each year. The International Diabetes Federation estimated that around 537 million individuals worldwide were living with diabetes in 2021, representing 10.5% of the global population. It is also projected that the number of people suffering from diabetes will increase to 783 million people in 2045.^1^ Diabetes mellitus (DM), especially type 2, is the most common type of diabetes worldwide, and it is often caused by an unhealthy lifestyle. This type of diabetes is commonly characterized by elevated blood glucose due to insulin resistance and impaired insulin secretion by pancreatic β-cells, leading to inadequate control of blood glucose levels.^2^ Prolonged impaired insulin control leads to the desensitization of cells and tissues to insulin stimulation, resulting in an inability to take up glucose from the blood. This condition results in chronic hyperglycemia, which involves various complex cellular and molecular mechanisms. Several mediators, such as dipeptidyl peptidase-4 (DPP-4), sodium–glucose cotransporters (SGLT), and the PI3K/Akt/mTOR pathway are strongly involved in diabetes progression.^3^, ^4^, ^5^, ^6^

The involvement of the enzyme DPP-4 in diabetes progression is related to its function as an inhibitor of incretin hormones, particularly glucagon-like peptide-1 (GLP-1) and glucose-dependent insulinotropic polypeptide (GIP). Both of these hormones act as substrates for DPP-4, which can degrade rapidly when they bind to it.^3^ Normally, incretins are secreted by enteroendocrine cells and rapidly elevated in response to food intake. These hormones then act as insulinotropic agents, inducing more insulin secretion to control the plasma glucose level.^7^ GLP-1 is known to directly inhibit glucagon secretion from α-cells as well as indirectly inhibiting glucagon by increasing insulin production, which is a natural inhibitor of glucagon. This combined mechanism leads to low glucagon levels, and thus lower plasma glucose levels.^8^^,^^9^ GLP-1 is also able to promote β-cell survival via various interconnected molecular mechanisms, mainly through binding to its receptor, which then activates further downstream signaling.^10^ The binding of GLP-1 to its receptor, which is a G-protein-coupled receptor, induces cAMP production, which then activates the CREB transcription factor to upregulate insulin receptor substrate 2 (IRS-2) and promote β-cell survival.^11^ The presence of DPP-4 impairs the function of incretin in controlling plasma sugar levels by directly inhibiting incretins, leading to decreased insulin production. Therefore, DPP-4 inhibitors, such as vildagliptin, sitagliptin, and gosogliptin, are among the primary treatments for type 2 DM, and often combined with other therapies such as metformin, insulin, or SGLT2 inhibitors.^12^^,^^13^

The use of SGLT2 inhibitors is based on the crucial role of SGLT2 in modulating glucose homeostasis. Glucose filtered from the blood in the glomerulus is reabsorbed into the bloodstream, particularly in the early section of the proximal tubule, mediated by SGLT2. SGLT2 is also responsible for 90% of glucose reabsorption into the bloodstream, making it an important mediator of glucose homeostasis.^14^^,^^15^ Diabetes is characterized by high plasma glucose levels, and thus SGLT2 constantly reabsorbs excess glucose into the bloodstream, resulting in persistently high glucose levels, further worsening the diabetic condition. Therefore, several SGLT2 inhibitors are used to block this protein, preventing glucose reabsorption into the bloodstream and helping lower plasma glucose levels.^16^^,^^17^ SGLT2 inhibitors, including canagliflozin, dapagliflozin, empagliflozin, and ertugliflozin, are frequently employed in clinical practice. These agents operate independently of β-cell function and insulin secretion, exerting their effects by inhibiting glucose reabsorption in the renal proximal tubules, thereby promoting glucosuria.^18^

Several known drugs and inhibitors can prevent the further worsening of diabetes, but the development of diabetes through multiple molecular pathways makes it challenging to design drugs that target them simultaneously. Thus, designing new drugs that target several molecular targets simultaneously is essential for further preventing the development of this disease.^19^ Among the various treatment options, the utilization of plant extracts for managing DM, particularly type 2 DM, has emerged as a promising therapeutic approach. Phytochemicals present in medicinal plant extracts are known to target multiple proteins associated with the pathogenesis of type 2 DM, thereby potentially preventing the progression and exacerbation of the disease.^20^^,^^21^ Polyherbal formulations of plant extracts are often employed to achieve better efficacy in treating type 2 DM,^22^ and this approach has also been tested on humans in various clinical trials.^23^

Plants with well-known antidiabetic properties include fenugreek (*Trigonella foenum-graecum*), paitan (*Tithonia diversifolia*), and turmeric (*Curcuma longa*). Various studies have investigated the antidiabetic potential of these plants, with positive results.^24^, ^25^, ^26^ However, previous studies have not utilized a combination of these three plant extracts with the aim of inhibiting proteins related to type 2 DM, especially DPP-4 and SGLT2. Information is also still scarce regarding the phytochemicals in these plants that are mainly responsible for their antidiabetic properties. The utilization of computational, or in silico, research is a crucial step in the drug discovery process, potentially enhancing the optimization of both novel and existing therapeutics targeting disease-specific proteins.^27^

Numerous studies have examined individual plants as antidiabetic agents, but few have investigated a combination of three herbs, i.e., fenugreek, paitan, and turmeric (FPT), using liquid chromatography-high resolution mass spectrometry (LC-HRMS) based metabolite profiling followed by multi-target computational screening. Therefore, this study aimed to provide a preliminary in silico evaluation of the effects of FPT-derived compounds against DPP-4 and SGLT2 as dual therapeutic targets, thereby contributing to a deeper understanding of potential antidiabetic mechanisms.

## Materials and Methods

### Extraction and identification of compounds in FPT

Polyherbal extract also known as the FPT formulation, was prepared from a combination of *Trigonella foenum-graecum* (fenugreek), *Tithonia diversifolia* (paitan), and *Curcuma longa* (turmeric). The plant materials were collected from UPT Laboratorium Herbal Materia Medica Batu, East Java, Indonesia (7.8677°S, 112.5193°E). The voucher specimen numbers for each plant are 250221.P G.208 (*Trigonella foenum-graecum*), 240614.PTN F MLG.597.228 (*Tithonia diversifolia*), and 240614.KNT L MLG.596.227 (*Curcuma longa*). The extraction procedure was performed according to the method described by Lestari et al.^28^ with modifications. First, 100 g each of powdered fenugreek, paitan, and turmeric was extracted with 1000 mL of water (1:10, w/v) and boiled at 95 °C for 5–10 min. The extract was then allowed to stand at room temperature, before filtering to separate the solid residues from the liquid extract. The liquid obtained from the filtrate was frozen at −20 °C for 24 h, followed by freeze drying. The resulting extracts were subsequently subjected to LC-HRMS analysis at the National Research and Innovation Agency (BRIN), Gunung Kidul, Yogyakarta, Indonesia. The LC-HRMS analytical conditions and procedures were adapted from those reported previously by Salsabila et al.^29^

### Characterization and profiling of bioactive compounds in FPT extract

Bioactive compounds in the FPT extract were characterized by drug-likeness analysis to evaluate the suitability of the compounds as drug candidates using the SwissADME web server. The parameters analyzed included drug-likeness, bioavailability score, and the solubilities of the compounds in water and lipids. All analyses were performed based on the SMILES data obtained for each compound from the PubChem database. Toxicity predictions were performed for each active compound using the ProTox 3.0 web server to classify toxicity levels based on LD_50_ values, as well as to evaluate potential organ toxicity, including hepatotoxicity, neurotoxicity, nephrotoxicity, respiratory toxicity, and cardiotoxicity. In addition, analysis of endpoint-based toxicity was performed, i.e., immunotoxicity and cytotoxicity. Furthermore, bioactivity analysis was conducted using the Way2Drug PASS Online web server. The analysis of bioactivities focused on activities related to diabetes, including antidiabetic activity, antioxidant activity, increased insulin sensitivity, and other mechanisms involved in regulating glucose levels in the body.

### Screening potential compounds using molecular docking

Bioactive compounds in the FPT formulation were identified through LC-HRMS profiling of the aqueous extract (See Table 1). Compounds were selected by data mining based on a best-match score >90%, and their three-dimensional structures and canonical SMILES data were retrieved from the PubChem database in SDF format for in silico analysis. Molecular docking was conducted against DPP-4 and SGLT2 using AutoDock Vina implemented in PyRx version 0.9.5.^30^^,^^31^ The crystal structures of DPP-4 (PDB ID: 3F8S) and SGLT2 (PDB ID: 7VSI) were obtained from the RCSB Protein Data Bank. Prior to docking, protein structures were prepared by removing water molecules and non-essential ligands using BIOVIA Discovery Studio 2021.^32^ The predicted active-site residues in SGLT2 were Arg336, Gly360, Val359, Asp454, Gly450, Ala672, Glu99, Lys321, and Leu283, and the predicted active-site residues in DPP-4 were Trp62, Arg125, Trp157, Glu205, Glu206, Ser209, Tyr547, Lys554, Tyr662, and Tyr666. The position and size of the grid box were validated using a re-docking method with a reference inhibitor, where a root mean square deviation (RMSD) value below 2 Å indicated a valid grid. Details of the molecular docking method are presented in Table 2. Binding affinities were evaluated based on the lowest predicted binding energies (kcal/mol), and the top-ranked compounds were subsequently selected for molecular dynamics simulations and visualized to elucidate key protein–ligand interactions.

**Table 1:** LC-HRMS profiling of bioactive compounds in FPT extract.

Name	Formula	Annot. DeltaMass [ppm]	Calc. MW	RT [min]	Area (Max.)	mzCloud Best Match
(+)-ar-turmerone	C_15_H_20_O	−1.68	216.15105	12.778	123529781.9	99.8
TEMPO	C_9_H_19_NO	−0.84	157.14653	8.932	14653743.41	90.9
β-carboline-3-carboxylic acid	C_12_H_12_N_2_O_2_	−1.07	216.08965	3.495	50441375.07	99.6
4-Coumaric acid	C_9_H_8_O_3_	−0.55	164.04725	5.319	63190828.57	94.3
4-Indolecarbaldehyde	C_9_H_7_NO	−0.82	145.05264	6.212	20341947.5	99.3
5-Hydroxymethyl-2-furaldehyde	C_6_H_6_O_3_	0.16	126.03171	1.696	50672968.08	96.5
Betaine	C_5_H_11_NO_2_	−0.31	117.07894	0.846	1344097047	93.5
Choline	C_5_H_13_NO	0.48	103.09976	0.812	551175910.3	92.3
Citric acid	C_6_H_8_O_7_	−3.32	192.02637	0.918	714464737.2	99.8
D-(+)-Pipecolinic acid	C_6_H_11_NO_2_	−1.16	129.07883	0.901	92345258.32	95.4
Gentisic acid	C_7_HO_4_	−4.65	154.02589	1.95	23316875.58	93.8
Gluconic acid	C_6_H_12_O_7_	−3.58	196.0576	0.827	16350570.5	94.8
Hexadecanamide	C_16_H_33_NO	−0.25	255.25615	14.297	10631824.73	97.5
Methyl isonicotinate	C_7_H_7_NO_2_	−1.51	137.04747	0.877	465928848.6	98
Nicotinic acid	C_6_H_5_NO_2_	0.01	123.03203	0.978	25528531.92	99.7
Oleamide	C_18_H_35_NO	−0.53	281.27172	14.711	59773013.81	95.2
trans-3-Indoleacrylic acid	C_11_H_9_NO_2_	−1.09	187.06312	2.209	160981512.6	93
α,α-Trehalose	C_12_H_22_O_11_	−0.63	342.116	0.791	575696828	97.8

**Table 2:** Molecular docking settings.

Protein	Reference Inhibitor	Protein Active Sites	Grid Coordinates		Re-docking RMSD (>2.0 Å)
			Center	Dimensions	
DPP-4 (PDB ID: 3f8s)	Gosogliptin (Pubchem ID: 11516136)	Trp62, Arg125, Trp157, Glu205, Glu206, Ser209, Tyr547, Lys554, Tyr662, Tyr666	x = 15.31152 y = 17.94062 z = 36.85483	x = 25.00000 y = 26.52096 z = 24.63747	1.427
SGLT2 (PDB ID: 7vsi)	Empagliflozin (Pubchem ID: 11949646)	Arg336, Gly360, Val359, Asp454, Gly450, Ala672, Glu99, Lys321, Leu283	x = 34.20245 y = 56.29991 z = 48.28213	x = 25.57120 y = 26.67014 z = 33.77019	1.215

### Molecular dynamics simulations

Compounds with the best binding affinity values were selected and subsequently prepared for molecular dynamics simulations. The simulations were performed using YASARA (Yet Another Scientific Artificial Reality Application) version 19.12.14 with the AMBER14 force field. Ligand parameterization was performed using the AMBER14 force field integrated in YASARA, where atom types and partial charges were automatically assigned. The resulting ligand topologies were then integrated into the protein–ligand complexes for molecular dynamics simulations. The simulation environment was set to physiological conditions: 37 °C, pH 7.4, 0.9% salt concentration, 1 atm pressure, and water density of 0.997 g/mL. Each simulation was run for 250 ns, with trajectory data automatically recorded every 125 ps using the md_runfast and md_bindingenergy macros. Post-simulation analyses included RMSD using the md_analyze program, root mean square fluctuation (RMSF) via md_analyzeres, and receptor–ligand binding energy calculation through md_bindingenergy, employing the molecular mechanics Poisson–Boltzmann surface area (MM/PBSA) method.^31^

### Principal component analysis (PCA) and dynamic cross-correlation matrix (DCCM) analysis

The conformational dynamics of the DPP-4 and SGLT2 complexes were analyzed using DCCM and PCA implemented in RStudio. Molecular dynamics trajectories generated by YASARA Structure (.sim) were converted using the md_convert.mcr macro with the following parameters: waterincluded = 0, srcformat = “sim”, dstformat = “xtc”, rotremoved = 0, and the AMBER14 force field. The resulting XTC files, corresponding to the native GROMACS trajectory format, were further converted using Visual Molecular Dynamics (VMD) to obtain DCD files compatible with NAMD.^33^ DCCM was constructed based on all Cα atoms from a 250 ns molecular dynamics simulation to assess correlated and anticorrelated domain motions. PCA was performed based on water-stripped XTC trajectories in conjunction with the corresponding protein structure files (PDB) using the mktrj.pca function in the Bio3D package.^34^ Eigenvalue and eigenvector analyses were subsequently conducted to identify the two principal components with the highest variance, providing insights into the dominant collective motions and thermodynamic stability of each complex.^35^^,^^36^

## Results and discussion

LC-HRMS profiling of the polyherbal FPT formulation successfully identified 18 compounds with best match scores above 90 (Table 1), indicating high confidence levels in structural identification. These compounds represented diverse phytochemical classes, including flavonoids, diterpenoids, alkaloids, and curcuminoids, which are known for their potential biological activities relevant to glucose metabolism and insulin regulation.

**Figure 1:**
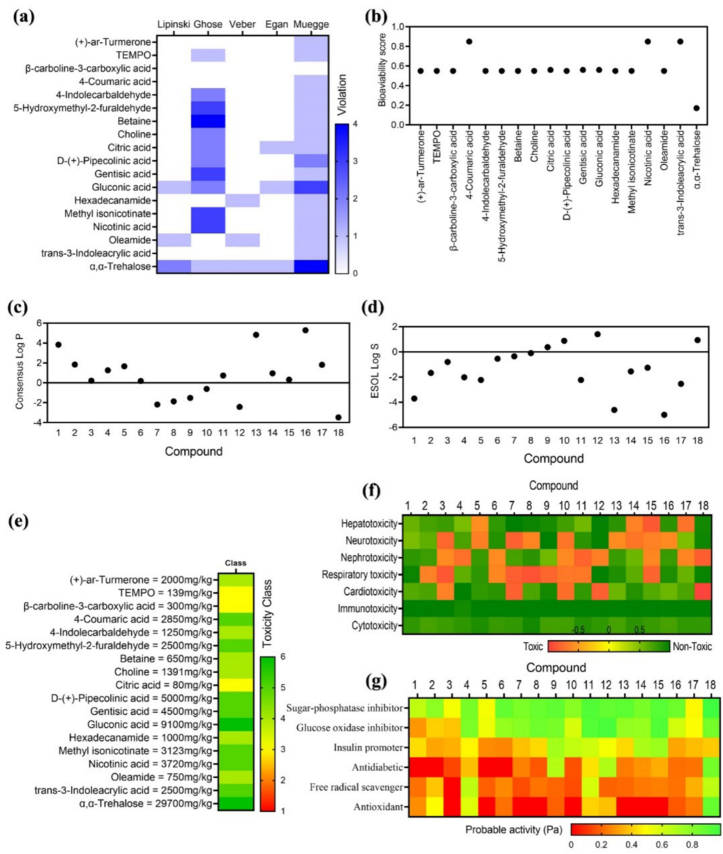
Characterization and profiling of bioactive compounds in FPT extract: (a) drug-likeness analysis, (b) bioavailability score, (c) consensus Log P representing lipophilicity of compound, (d) estimated solubility representing aqueous solubility, (e) toxicity classes and LD_50_ values, (f) toxicity effects, and (g) biological activity.

According to drug-likeness analysis, the majority of the compounds were found to have potential as drug candidates, and this was supported by evaluation parameters based on Lipinski's, Veber's, and Egan's rules, where most compounds met the criteria with minimal violations. This was also supported by the relatively good bioavailability values (≥0.5) for nearly all compounds, which reinforced their potential. In addition, α,α-trehalose, hexadecanamide, and oleamide had relatively lower drug-likeness profiles compared with the other compounds. This limitation was primarily influenced by water solubility and lipophilicity parameters, implying the low solubility and distribution of the compounds in biological systems, thereby limiting their potential as drug candidates. In addition, the toxicity ranges of the compounds present in the FPT extract indicated that they are safe for consumption, with a varied range of LD_50_ doses. Overall, the compounds exhibited non-toxic activities based on their immunotoxicity and cytotoxicity profiles, although long-term use of the extract should still be evaluated, particularly for individuals with respiratory, nephrotoxicity, and neurotoxicity issues. Furthermore, the probable activities of compounds in the FPT extract indicated a high potential for regulating blood glucose levels based on their roles as sugar-phosphatase inhibitors and glucose oxidase inhibitors (See Figure 1).

To explore their pharmacological potential, all 18 identified compounds were subjected to molecular docking against two key therapeutic targets associated with glucose homeostasis: SGLT2 and DPP-4. These targets were selected based on their clinically validated roles in modulating blood glucose levels and as established targets in current antidiabetic drug development. Docking analysis was conducted to identify putative candidate compounds with potential dual or selective inhibitory activities against SGLT2 and DPP-4, which may act synergistically with the intrinsic bioactive mechanisms associated with the herbal extracts. The integration of LC-HRMS based metabolite profiling with in silico molecular docking provides a systematic and rational strategy for identifying promising bioactive candidates from complex herbal matrices, thereby supporting the development of plant-based therapeutics for diabetes management.

**Figure 2:**
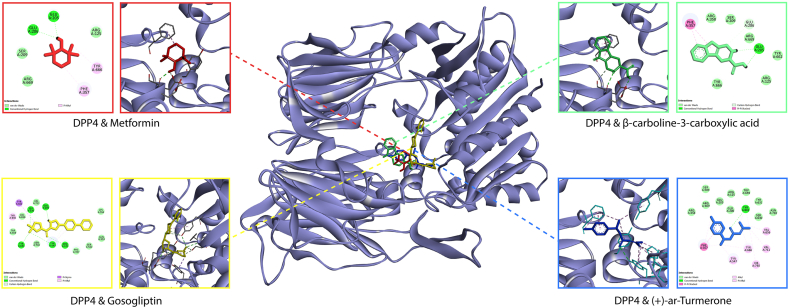
Molecular docking visualization of potential compound in the DPP4 binding site.

**Figure 3:**
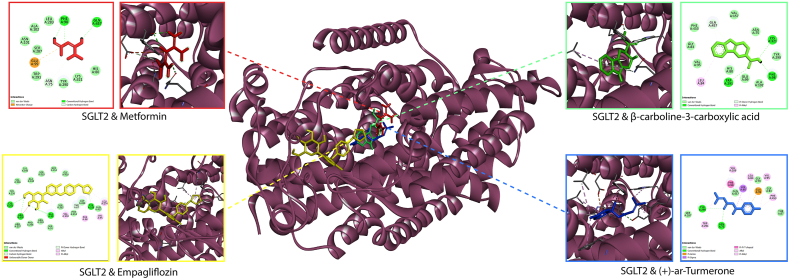
Molecular docking visualization of potential compound in the SGLT2 binding site.

Based on molecular docking analysis, β-carboline-3-carboxylic acid and (+)-ar-turmerone were identified as compounds with moderate binding affinities toward DPP-4 and SGLT2 (Figure 2 and Figure 3), and their binding profiles were also compared to those for reference compounds, including metformin, empagliflozin, and gosogliptin (See Table 3). The binding affinity of metformin to DPP-4 was relatively weak, and characterized by few hydrogen bonds and hydrophobic interactions, indicating a low binding affinity. This aligns with the pharmacological mechanism of metformin, which does not involve direct DPP-4 inhibition but instead it maintains the plasma glucose level through alternative pathways.^37^^,^^38^ Gosogliptin exhibited the strongest binding interactions with DPP-4, forming numerous hydrogen bonds and hydrophobic contacts, consistent with its established clinical role as a DPP-4 inhibitor.^39^ β-Carboline-3-carboxylic acid had a favorable binding affinity, with significant interactions, including multiple hydrogen bonds and hydrophobic contacts, where the binding affinities ranged from strong to moderate. Therefore, this compound may exhibit a moderate binding affinity toward DPP-4 based on the docking results. In addition, (+)-ar-turmerone mainly interacted with DPP-4 through hydrophobic interactions, forming fewer hydrogen bonds than gosogliptin and β-carboline-3-carboxylic acid. These interactions suggest a weaker binding profile compared with the reference inhibitor because previous studies indicate that changes in interaction numbers and shifts from hydrogen bonds to other interactions could influence the modulation of the targeted protein.^40^ Overall, gosogliptin had the highest binding affinity, followed by β-carboline-3-carboxylic acid, and (+)-ar-turmerone had a comparatively lower interaction strength. Compared with the other compounds, metformin had a weak binding activity. However, although (+)-ar-turmerone exhibited a moderate docking affinity toward DPP-4, this result was not supported by MM/PBSA analysis, which yielded a positive binding free energy (+53.907 kJ/mol), indicating an unfavorable interaction, suggesting that the compound may not form a stable complex with DPP-4 under dynamic conditions. Importantly, the positive binding energy determined by MM/PBSA for the DPP-4 complex indicated a thermodynamically unfavorable interaction, and thus (+)-ar-turmerone is unlikely to act as a stable DPP-4 inhibitor under physiological conditions. This finding contradicts the docking prediction and highlights the importance of dynamic simulation for refining initial screening results.

**Table 3:** Molecular docking results.

No	Name	Pubchem ID	3f8s	7vsi
1	Gosogliptin/empagliflozin	11516136/11949646	−8.7	−8.6
2	β-carboline-3-carboxylic acid	98285	−6.8	−8.6
3	(+)-ar-turmerone	160512	−6.9	−8.3
4	trans-3-Indoleacrylic acid	5375048	−6.6	−8
5	α,α-Trehalose	7427	−6.4	−7.7
6	4-Coumaric acid	637542	−5.6	−7.2
7	4-Indolecarbaldehyde	333703	−5.3	−7.1
8	Gentisic acid	3469	−5.6	−6.9
9	Oleamide	5283387	−5.5	−6.7
10	Hexadecanamide	69421	−5.3	−6.5
11	TEMPO	549976	−4.9	−6.3
12	Citric acid	311	−6	−6.1
13	Nicotinic acid	938	−5.2	−6
14	D-(+)-Pipecolinic acid	736316	−5.1	−6
15	Gluconic acid	10690	−5.8	−5.8
16	Methyl isonicotinate	227085	−5.2	−5.6
17	5-Hydroxymethyl-2-furaldehyde	237332	−5	−5.5
18	Betaine	247	−3.7	−4.1
19	Choline	305	−3.2	−4
20	Metformin	4091	−5	−5.4

Molecular docking analyses were also performed to investigate the interactions between SGLT2 and various ligands, including metformin, empagliflozin, β-carboline-3-carboxylic acid, and (+)-ar-turmerone. The results are depicted in Figure 3. Metformin exhibited weak interactions with SGLT2, characterized by limited hydrogen bonding with polar residues and minimal hydrophobic interactions, thereby correlating with its low binding affinity and consistent with its pharmacological mechanism, which does not involve the direct inhibition of SGLT2. Metformin canonically modulates the plasma glucose level, involving AMPK activation in the liver, skeletal muscle, and gut, reducing gluconeogenesis and improving intestinal glucose handling.^38^^,^^41^ Thus, the functions of metformin differ from those of SGLT2, which modulates glucose levels in the kidney.^42^ Empagliflozin had the strongest and most stable interaction with SGLT2. This ligand exhibited a high affinity for the SGLT2 binding pocket, establishing multiple hydrogen bonds and hydrophobic interactions. These strong and stable interactions support the role of empagliflozin as an SGLT2 inhibitor, and empagliflozin is utilized clinically in the management of type 2 DM.^43^ In addition, β-carboline-3-carboxylic acid had a significant interaction with SGLT2. Several hydrogen and hydrophobic interactions were identified, supporting the idea that this compound could be a potential SGLT2 inhibitor. However, (+)-ar-turmerone exhibited a weaker interaction with SGLT2, mainly forming hydrophobic interactions with fewer hydrogen bonds. Several previous studies indicate that hydrogen bonds have a more substantial effect than hydrophobic interactions, which usually result in only a partial effect on the targeted protein.^44^^,^^45^ Therefore, the interaction between (+)-ar-turmerone and SGLT2 was predicted to be weaker than those with empagliflozin and β-carboline-3-carboxylic acid, which also suggests that (+)-ar-turmerone may only partially modulate the activity of SGLT2. These results indicate that several compounds could serve as promising alternative SGLT2 inhibitors for development.

**Figure 4:**
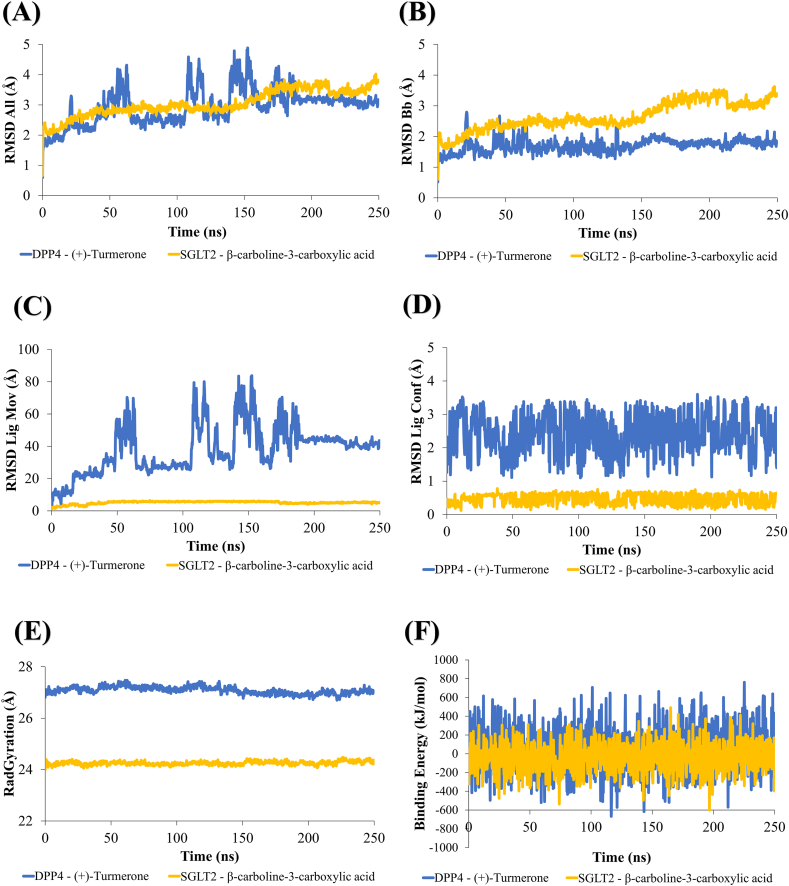
Molecular dynamics simulation of potential compound during 250 ns trajectory: (a) RMSD for the protein–ligand complex, (b) RMSD for the protein backbone, (c) RMSD for ligand movement, (d) RMSD for ligand conformation, (e) radius of gyration, and (f) binding free energy profile based on MM/PBSA analysis.

In addition to molecular docking, molecular dynamics analyses were performed as further refinement and validation tools. Molecular dynamics analysis mainly captures the conformational diversity of a protein after ligand binding in real-time and that is most likely to occur under physiological conditions.^46^ According to the results obtained by molecular dynamics analysis based on the complex RMSD value (RMSD all) over 250 ns (Figure 4), the interaction between the potential compound with SGLT2 exhibited relatively more favorable interaction behavior compared with DPP-4 in the first 100 ns. These fluctuations suggest that the complexes exhibited dynamic behavior rather than highly stable binding. The DPP-4 complex exhibited fluctuations over several time ranges, specifically around 50, 100, 150, and near 200 ns. The RMSD backbone value indicated that the DPP-4 protein structure was relatively more stable during 250 ns in the simulation, and that the SGLT2 protein structure fluctuated after 150 ns. RMSD ligand movement analysis of the ligand inside the DPP-4 complex indicated great fluctuation from the start of the simulation until the end. Conversely, the ligand in the SGLT2 complex had an RMSD value below 3 Å during the first 10 ns. The use of an RMSD value below 3 Å to determine a stable protein conformation is also supported by several previous studies that treated an RMSD value below 3 Å as indicative of a stable protein conformation.^47^^,^^48^ The average RMSD ligand movement values were 38.089 Å for the DPP-4 complex and 5.14 Å for SGLT. The average conformation value for the SGLT2 complex was 0.465 Å, which was lower than that for DPP-4 (2.419 Å at 250 ns). These results suggest that the ligand in the SGLT2 complex exhibited relatively more consistent positioning compared with DPP-4. The radius of gyration also exhibited relatively stable fluctuations during the simulation, indicating no significant change in the protein structure. The binding energy calculation also showed that the SGLT2 complex had a value of −42.135 kJ/mol, and that for the DPP-4 complex was 53.907 kJ/mol. There is no universal threshold for a good binding energy but a previous study found that more negative values indicate better results.^49^ Thus, the potential compound had a stronger and more stable binding affinity to SGLT2 than DPP-4.

RMSF indicates the movement for each residue or atom in a protein over time. The molecular simulation for DPP-4 indicated fluctuation in the Glu244 residue, whereas the highest peak for SGLT2 was at the Asp21 residue, reaching 10 Å. SGLT2 also had higher values at several residues, such as Arg49, Trp172, Ala247, Arg416, Gln448, and Glu641, compared with other residues. RMSF analysis revealed notable fluctuations, with peaks reaching up to 10 Å in the SGLT2 complex, indicating significant local flexibility. These fluctuations suggest that certain protein–ligand complexes are dynamic and the overall binding stability may be reduced. A previous study also found that RMSF values not exceeding 2 Å still indicated good stability.^50^

**Figure 5:**
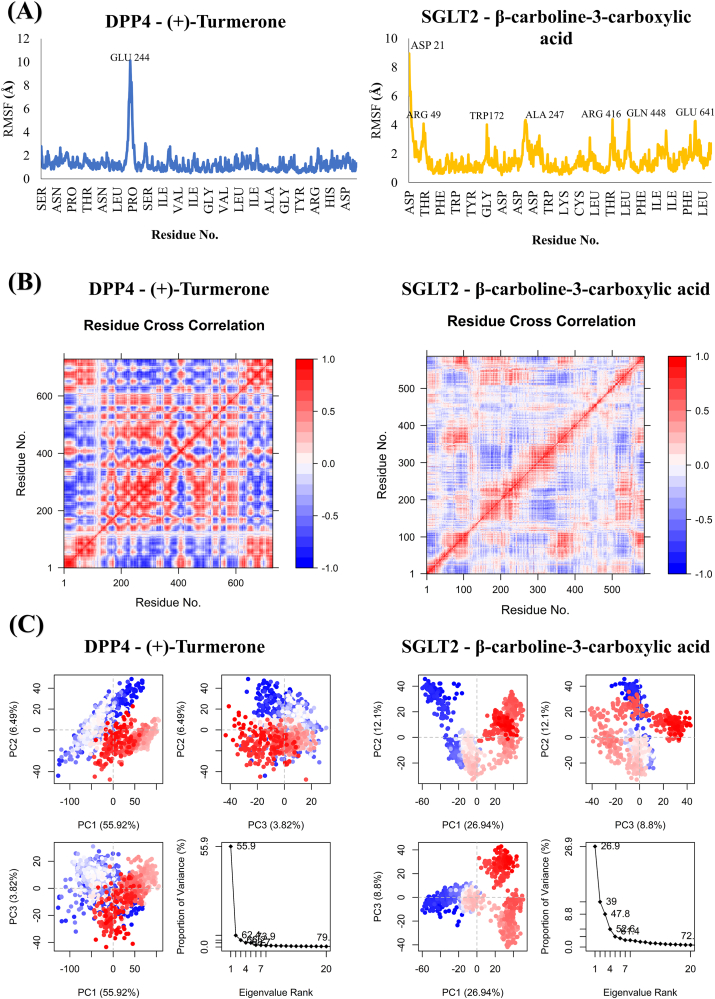
Molecular dynamics–based analyses of DPP4 and SGLT2: (a) root mean square fluctuation (RMSF), (b) dynamic cross-correlation matrix (DCCM), and (c) principal component analysis (PCA).

DCCM was further employed to identify the correlated and anti-correlated movements of residues after ligand binding to the targeted protein.^51^ The DCCM results for the DPP-4 protein indicated that amino acid residues moved in a compact, coordinated manner, as shown in red on the map in Figure 5. These positive correlations show that most parts of the protein moved together in a stable, strong DPP-4 complex conformation. The blue area reflects opposite movements in a particular domain. However, the dominant red area indicates that the DPP-4 molecule tended to maintain structural compactness during its interaction with the ligand. The DCCM results with the SGLT2 protein indicated a varied correlation pattern between residues. The red color observed diagonally and in some areas indicated that many residue pairs moved synchronously and the patterns reflected differences in internal dynamics between the systems but not necessarily stronger binding stability. In addition, the blue area indicates residues that moved in the opposite direction, highlighting the flexibility and dynamics of the SGLT2 structure during the simulation. The molecular interaction of the protein tended to have a compact conformation, consistent with the observed stability and interaction strength of the complex, as indicated by the red regions with correlation values close to +1.

Applying PCA to molecular dynamics trajectory data can simplify the complexity of atomic motions and clarify the main collective modes that influence a system's dynamics.^52^ The eigenvalues represent the amount of variance in atomic movements attributable to each principal component. For the DPP-4 protein, the combined variance explained by PC1 and PC2 was 62.41%, indicating that these two components were responsible for a significant part of the structural fluctuations, suggesting that the system exhibited a more directed and stable movement pattern, which aligned with a tendency toward more stable ligand–target interactions. Conversely, for the SGLT2 protein, the cumulative variance explained by PC1 and PC2 was 39.04%, implying that atomic movements were more dispersed and complex, resulting in a relatively lower degree of conformational stability. Both the DCCM and PCA results aligned with other parameters, such as the RMSD, RMSF, and binding energy. Collectively, these findings strengthen the conclusion that the ligand-SGLT2 complex exhibited better dynamical stability compared with the ligand-DPP-4 complex. Overall, the molecular dynamics results suggested that the complexes exhibited dynamic behavior, and the observed fluctuations indicated that the binding stability was moderate rather than strong.

To provide a biological context, the potential involvements of SGLT2 and DPP-4 in glucose regulation pathways are discussed in the following based on previous studies; however, these interactions were not directly investigated in the present study and should be considered hypothetical. Studies indicate that SGLT2 and DPP-4 proteins are potentially interconnected and strongly influence the modulation of glucose levels through the incretin axis, as reflected by the high betweenness values for GLP1R and glucagon, which can be explained by SGLT2 acting through glucagon encoded by the proglucagon precursor, and it is subsequently processed in intestinal L-cells to produce GLP-1 and GLP-2.^8^^,^^53^ DPP-4 functions as a natural inhibitor of GLP-1 by degrading this incretin hormone, which would otherwise bind to GLP1R to stimulate insulin secretion; consequently, the presence of DPP-4 attenuates GLP-1 mediated signaling.^3^ By contrast, some previous studies have shown that SGLT2 inhibitors increase GLP-1 secretion in animal models, possibly through enhanced urinary glucose excretion following SGLT2 inhibition.^54^ Through interacting with glucagon, SGLT2 inhibition may further regulate blood glucose levels by modulating insulin and glucagon signaling pathways.^55^ SGLT2 is known to indirectly interact with GLP1R and glucagon, but the precise molecular mechanisms involved in these interactions remain unclear and warrant further investigation. The findings obtained in the present study were based on an in silico computational approach, which is a limitation. Therefore, further investigations involving in vitro and in vivo studies are necessary to validate these results, particularly for assessing the biological activities of FPT-derived bioactive compounds against DPP-4 and SGLT2.

## Conclusions

The present preliminary study yielded computational insights into the potential interactions of FPT-derived compounds with SGLT2 and DPP-4 as antidiabetic treatment targets, where (+)-ar-turmerone and β-carboline-3-carboxylic acid exhibited differential binding behaviors toward DPP-4 and SGLT2. β-Carboline-3-carboxylic acid had favorable interactions with SGLT2, whereas (+)-ar-turmerone exhibited only a moderate docking affinity toward DPP-4, which was not supported by the MM/PBSA results, indicating unfavorable binding. Comprehensive computational analyses confirmed the superior binding stability and thermodynamic favorability toward SGLT2 compared with DPP-4 based on integrated molecular docking, dynamics simulations, free energy analyses, DCCM, and PCA. However, these findings were obtained based only on in silico approaches and should be interpreted with caution. Further experimental validation, including in vitro and in vivo studies, is required to confirm their biological activities and therapeutic relevance.

## Ethical approval

The authors declare that there are no ethical concerns related to this study.

## Authors contributions

GSP: Conceptualization, Validation, Supervision, Resources, Methodology, Investigation, Funding acquisition, Writing – original draft, Writing – review & editing. API: Conceptualization, Visualization, Software, Methodology, Investigation, Formal analysis, Data curation, Writing – review & editing. NDL: Conceptualization, Software, Methodology, Investigation, Formal analysis, Data curation, Writing – review & editing. All authors have critically reviewed and approved the final draft and are responsible for the content and similarity index of the manuscript.

## Source of funding

This work was supported by the 10.13039/501100002385Ministry of Higher Education, Science, and Technology of Republic of Indonesia (Kemdiktisaintek RI) [grant numbers 017/LL7/10.13039/100001588DT.05.00/PL/2025, E.5.c/122.02/PNL/LPPM-10.13039/501100021259UMM/V/2025].

## Conflict of interest

The authors have no conflicts of interest to declare.
